# Identification of early predictive biomarkers for severe cytokine release syndrome in pediatric patients with chimeric antigen receptor T-cell therapy

**DOI:** 10.3389/fimmu.2024.1450173

**Published:** 2024-09-12

**Authors:** Meng Su, Luoquan Chen, Li Xie, Aurore Fleurie, Renaud Jonquieres, Qing Cao, Benshang Li, Ji Liang, Yanjing Tang

**Affiliations:** ^1^ Department of Hematology/Oncology, National Health Committee Key Laboratory of Pediatric Hematology & Oncology, Shanghai Children’s Medical Center, Shanghai Jiao Tong University School of Medicine, Shanghai, China; ^2^ Shanghai Children’s Medical Center–bioMérieux Laboratory, Shanghai Children’s Medical Center, Shanghai Jiao Tong University School of Medicine, Shanghai, China; ^3^ bioMérieux (Shanghai) Company Limited, Shanghai, China; ^4^ Open Innovation & Partnerships Department, bioMérieux SA, Marcy l’Etoile, France; ^5^ Infectious Disease Department, Shanghai Children’s Medical Center, Shanghai Jiao Tong University School of Medicine, Shanghai, China

**Keywords:** CAR-T cell therapy, cytokine release syndrome (CRS), biomarker, early prediction, decision tree model

## Abstract

CAR-T cell therapy is a revolutionary new treatment for hematological malignancies, but it can also result in significant adverse effects, with cytokine release syndrome (CRS) being the most common and potentially life-threatening. The identification of biomarkers to predict the severity of CRS is crucial to ensure the safety and efficacy of CAR-T therapy. To achieve this goal, we characterized the expression profiles of seven cytokines, four conventional biochemical markers, and five hematological markers prior to and following CAR-T cell infusion. Our results revealed that IL-2, IFN-γ, IL-6, and IL-10 are the key cytokines for predicting severe CRS (sCRS). Notably, IL-2 levels rise at an earlier stage of sCRS and have the potential to serve as the most effective cytokine for promptly detecting the condition’s onset. Furthermore, combining these cytokine biomarkers with hematological factors such as lymphocyte counts can further enhance their predictive performance. Finally, a predictive tree model including lymphocyte counts, IL-2, and IL-6 achieved an accuracy of 85.11% (95% CI = 0.763–0.916) for early prediction of sCRS. The model was validated in an independent cohort and achieved an accuracy of 74.47% (95% CI = 0.597–0.861). This new prediction model has the potential to become an effective tool for assessing the risk of CRS in clinical practice.

## 1 Introduction

In recent years, CAR-T cell therapy has revolutionized the treatment of relapsed/refractory (R/R) B-cell malignancies, achieving unprecedented responses, especially in B-cell acute lymphocytic leukemia (B-ALL) and B-cell non-Hodgkin lymphoma (B-NHL) (1–3). Reported complete remission (CR) rates are as high as 83% in R/R B-ALL along with overall response rates of 54% in R/R B-NHL. Currently, the FDA has approved CAR-T cell therapy for treating certain children and young adults with ALL. Although CAR-T cell therapy has yet to be FDA approved in other pediatric cancers, treatment of B-NHL, mantle cell lymphoma, follicular lymphoma, and multiple myeloma are approved in ages 18 years and older and clinical trials are ongoing for other pediatric cancers (4, 5).

Although CAR-T cell therapy has demonstrated remarkable efficacy in treating patients with R/R B cell malignancies, adverse events associated with this therapy serve as a significant barrier to treatment and can even result in fatalities. Cytokine Release Syndrome (CRS) is the most common adverse event, with an incidence of approximately 54% to 91% and severe CRS (sCRS) rates range from 8.3% to 43% (6–8). To provide a uniform consensus grading system for CRS, the American Society for Transplantation and Cellular Therapy (ASTCT) has published a consensus scale for grading the severity of CRS based on clinical symptoms, which ranges from grade 1 (mild) to grade 5 (fatal) (9). CRS often becomes life-threatening from grade 3 onwards and is known as sCRS, necessitating prompt medical interventions and the use of IL-6 blockers such as tocilizumab (10, 11). The ASTCT consensus grading scale for CRS is based on the severity of clinical symptoms. However, the variability of clinical symptoms and differing patient perspectives make it challenging to accurately grade CRS. Thus, specific biomarkers are needed to help for grading, monitoring and effectively treating CRS.

CRS arises as a direct consequence of overactivation of the immune system, which leads to a substantial increase in various serum cytokines, such as interleukin-6 (IL-6), interferon gamma (IFN-γ), granulocyte-macrophage colony-stimulating factor (GM-CSF), interleukin-1 (IL-1), or monocyte chemoattractant protein 1 (MCP-1) (6, 12–16). IL-6 is regarded as the principal cytokine driving CRS, instigating a proinflammatory signaling cascade that underpins several sCRS mainstay symptoms (17). CRS may share clinical and pathophysiological similarities with systemic inflammatory response syndrome, as well as macrophage activating syndrome/hemophagocytic lymphohistiocytosis. Thus, measuring and analyzing immune factors may aid in predicting sCRS and even help differentiate CRS and other complications including infection or sepsis following CAR-T treatment (18).

Here, we report the clinical and laboratory findings from 95 pediatric patients with R/R B-ALL or B-NHL who received lymphodepletion chemotherapy followed by coadministration of CD19- and CD22- targeted CAR-T cells (19, 20). We investigated the differential expression of various immune factors in pediatric patients before and after CAR-T cell infusion. Through this, we aimed to identify immunologic signatures that indicate the occurrence/risk of sCRS and provide direct and reliable indicators for clinicians to manage CRS.

## 2 Materials and methods

### 2.1 Study design and population

We enrolled patients with relapsed/refractory CD19+ and CD22+ B-ALL or B-NHL (Burkitt lymphoma, Diffuse large B-cell lymphoma, lymphoblastic lymphoma, primary mediastinal B-cell lymphoma, High-grade B-cell lymphoma) in a phase II clinical trial (Chinese Clinical Trial Registry: ChiCTR2000032211) that evaluated the safety and efficacy of coadministration of CD19- and CD22- targeted chimeric antigen receptor (CAR) T cells therapy in childhood B-ALL or B-NHL. All patients were enrolled based on the specified inclusion and exclusion criteria detailed in the **Supplementary Appendix 1**. Briefly, pediatric patients with relapsed or refractory hematological malignancies were eligible for inclusion, excluding those with severe comorbidities or active infections that could potentially affect their eligibility or ability to complete the study. The study was approved by the ethics committee of Shanghai Children’s Medical Center. Written informed consent were signed by the guardians or patients. In total, clinical and laboratory data were collected from 56 patients with R/R B-ALL and 39 patients with R/R B-NHL who underwent CAR-T cell therapy in the training cohort and 47 patients with R/R B-ALL who underwent CAR-T cell therapy in the validation cohort at Shanghai Children Medical Center.

### 2.2 Lymphodepletion chemotherapy, CAR-T cell manufacture, and infusion

**Figure 1:**
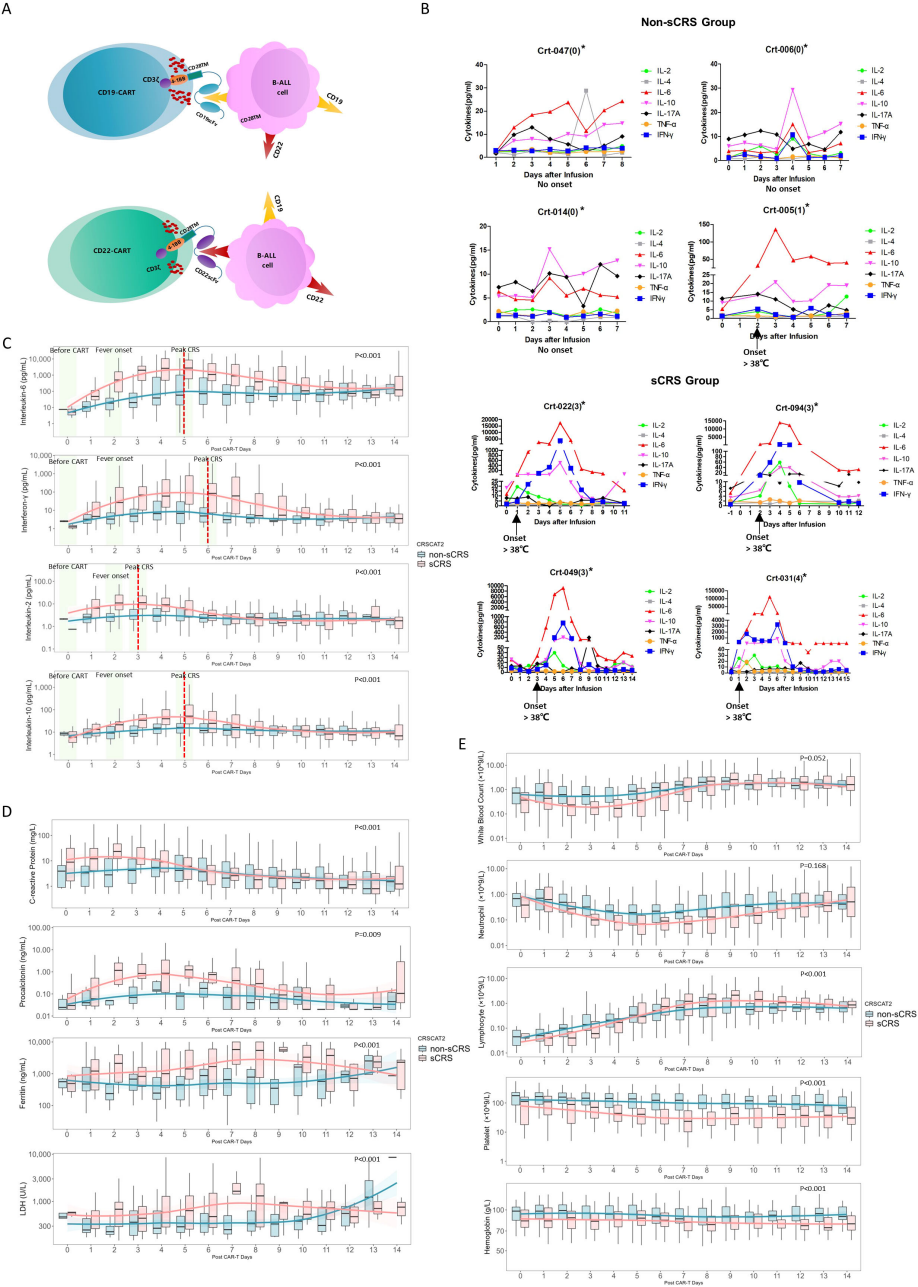
Cytokine expression patterns in non-sCRS group and sCRS group. **(A)** Diagram of CAR-T construct targeting for CD19 and CD22 expressing B cell malignancies. **(B)** Concentrations of seven released cytokines in serum obtained from individual patients at the indicated time points. *The Crt-nnn represents the code number of individual patient and the number in parentheses indicates the CRS Grading of the patient. **(C-E)** Concentrations of indicated biomarkers in patients at the indicated time points, including **(C)** four released cytokines, **(D)** four conventional markers and **(E)** five hematological markers. The red dashed lines indicated the peak value day of the sCRS group, while the three pivotal time windows: before CAR-T cell infusion, fever onset, and peak CRS, are highlighted in a light green area.

Lymphodepletion chemotherapy consisted of fludarabine (30-40 mg/m^2^ × 3 days) administered on Days -4 to -2 and cyclophosphamide (500 mg/m^2^ × 2 days) on Days -4 and -3. The design of the CAR transgene (as illustrated in **Figure 1A**) and CAR-T cell manufacturing have been described previously (19). Briefly, within 3 days of eligibility, CD3+ T lymphocytes were harvested from peripheral blood (1-2 mL/kg) and processed at the Shanghai Children’s Medical Center to produce CAR-T cells. After Ficoll-Hypaque centrifugation, anti-CD3 Microbead sorting, and 24-48 hours of stimulation with anti-CD3/CD28 beads, T cells were transduced with CD19- or CD22-specific CAR lentiviral vectors containing 4-1BB costimulatory and CD3 zeta domains. Prior to administration, CAR-T cells underwent rigorous quality tests, including viability assessment by Trypan blue, CAR-T cell expression analysis by flow cytometry using BD FACS Canto II (BD Biosciences), and cytotoxicity validation through co-culture with GFP+REH target cells. These tests ensured the efficacy and safety of the CAR-T cell product. The CD19- and CD22-specific CAR-T cells were cultured separately and then pooled together at a ratio of 1:1, washed, resuspended in saline solution with 2.5% human serum albumin, and transported to the medical center where the patient received the infusion at a dose between 1.66 × 10^6^ and 6.82 × 10^6^ cells/kg on day 0.

### 2.3 CRS grading and neurotoxicity

The severity of CRS and the immune effector cell-associated neurotoxicity syndrome (ICANS) was graded according to the ASTCT consensus scale (9). Non-sCRS group was defined as patients with no CRS, grade 1 and grade 2. sCRS group included patients who exhibited CRS symptoms ranging from grade 3 to grade 5. Similarly, non-severe ICANS (sICANS) group was defined as patients who did not develop ICANS or had grade 1 to 2 ICANS symptoms. sICANS group comprised patients who experienced ICANS symptoms ranging from grade 3 to grade 5.

### 2.4 Biomarker evaluation

We collected clinical and laboratory data on the included 95 patients. Peripheral blood was collected before CAR-T cell infusion, followed by daily collections for a period of two weeks post CAR-T therapy. To monitor immune status of patients undergoing CAR-T therapy, seven released cytokines (CD*7), including IL-2, IL-4, IL-6, IL-10, IL-17A, TNF-α, and IFN-γ were tracked using flow cytometry (Cytometric Bead Array Human Th1/Th2/Th17 Cytokine Kit, BD) to assess their predictive capacity for sCRS. Additionally, four biochemical markers (C-reactive protein [CRP], procalcitonin [PCT], lactate dehydrogenase [LDH] and Ferritin) and five hematological factors (Counts of white blood cell [WBC], Lymphocyte, Neutrophil, Hemoglobin, Platelet) were also analyzed to identify the optional combination for predicting sCRS.

### 2.5 Definitions of time windows for analysis

The data was analyzed using three different time windows, which were selected for distinct application scenarios: (1) Before CAR-T cells infusion: variables were analyzed using the closest test done within one month prior to CAR-T cell infusion to identify biomarkers that could predict the risk of developing sCRS before administering CAR-T cell therapy. (2) Fever onset: variables were analyzed on the day of fever onset or the day after fever onset if values were not available for that day. For patients without fever, values were selected and analyzed to coincide with the median day of fever in the fever group, which was around 4 days following CAR-T cell infusion. (3) Peak CRS: this analysis model aims to examine the highest value observed for each variable during the entire CRS duration.

### 2.6 Statistical analysis

For descriptive analysis, mean and standard deviation, median and interquartile range were used for continuous variable depends on normality test, as appropriate. Also, count and percentage were used for categorical variables. Normality of data were tested by Shapiro-Wilk test and QQ plot.

When comparing two groups, Student’s t-test or Mann–Whitney U test was used in continuous variables after checking on data normality, as appropriate. Categorical variables were compared between groups with the use of a chi-square test. When there were small numbers of categorical counts (e.g., less than 5), p-values were calculated using Fisher’s exact test. Repeat measurement of mixed model were conducted to compare between group difference of the profiling of each biomarker during study period. Two-dimensional principal component analysis was utilized to depict the distribution of all biomarkers between the sCRS and non-sCRS groups.

To evaluate the performance of each biomarker, the elastic net regression models were conducted to select biomarkers that predict incidents of sCRS. The predictive accuracy is assessed by successively fitting the model leaving 1 individual out, and the percent of individuals who were correctly classified by the model fit without their data are the leave one out cross-validation (LOOCV) predictive accuracy statistic (21).

Permutation feature importance was calculated to evaluate the relative importance of the biomarkers for prediction, which first ranks the biomarkers according to how much predictive accuracy would be lost by the elastic net model if the values of that variable were permuted; the incremental gains in accuracy for the model are then calculated as the variables are added 1 at a time according to this ranking (22). Also, the received operator curve (ROC) was used and corresponding area under curve (AUC)was calculated. All point estimation and corresponding 95% confidence interval (CI) for AUC were provided. The forest plot is employed to illustrate the AUC along with its standard deviation (SD) for each biomarker. A sCRS predictive model among CAR-T therapy was generated by decision tree model and discrimination performance of the models was evaluated using the concordance statistic (23). All tests are performed using R software (version 4.3.1) and python software (version 3.10.10). A two-sided p value less than 0.05 were considered as statistical significance.

## 3 Results

### 3.1 Clinical description of patients

**Table 1** provides the clinical information for the included patients. Of the 95 included patients, 59 developed non-sCRS (Grade 0-2) and 36 patients developed sCRS (Grade 3-5). Among the 95 patients, 56 were diagnosed with B-ALL and 39 were diagnosed with B-NHL. The age of the total cohort was 8.07 ± 4.32 years. There were no significant differences in age, gender, and leading cause for admission between the non-sCRS and sCRS groups. However, the bone marrow blasts before CAR-T cells infusion in the sCRS group was significantly higher than that in the non-sCRS group (5.8% vs. 0.9%), indicating that patients with higher tumor burden might be at higher risk of developing sCRS. Moreover, the median day from admission to fever occurrence was 1 day for sCRS patients, which was significantly earlier than that of non-sCRS patients (3 days). Consistently, the sCRS group experienced CRS earlier than the non-sCRS group (1, 0-1 day vs 3, 1.75-4 days). Additionally, the fever duration of the sCRS group was longer than that of the non-sCRS group (5 days vs. 4 days). And the CRS duration of the sCRS group was also longer than that of the non-sCRS group (5.5 days vs. 4 days). Furthermore, the sCRS group received more immune therapies (corticosteroids, tocilizumab, vasoactive medications) as well as ventilation following CAR-T treatment. Immune effector cell–associated neurotoxicity syndrome (ICANS) is another important complication that can occur following CAR-T therapy. In this study, it was found that eight patients (8.42%) developed severe ICANS, three from non-sCRS group and five from sCRS group.

**Table 1:** Baseline characteristics between different CRS groups.

Variable	All Participants*^1^*	CRS Grading*^1^*		Statistic	*P^2^*
		Grade, non-sCRS (n = 59)	Grade, sCRS (n = 36)		
Age (Years)	95 (8.07 ± 4.32)	59 (8.59 ± 4.25)	36 (7.21 ± 4.36)	1.52	0.132
Gender					0.465
*FEMALE*	23 (24.21)	16 (27.12)	7 (19.44)		
*MALE*	72 (75.79)	43 (72.88)	29 (80.56)		
Leading Cause for Admission					0.285
*ALL*	56 (58.95)	32 (54.24)	24 (66.67)		
*NHL*	39 (41.05)	27 (45.76)	12 (33.33)		
*Bone marrow blasts before CAR-T (%)*	*56 (2.05, 0 - 15.38)*	*32 (0.9, 0 - 5.1)*	*24 (5.8, 0.5 - 26)*	*265.5*	*0.047*
Treatment Type of First CAR-T					0.555
*allo-CAR-T*	3 (3.16)	1 (1.69)	2 (5.56)		
*auto-CAR-T*	92 (96.84)	58 (98.31)	34 (94.44)		
Cell Dose of the First CAR-T	95 (4.39 ± 2.41)	59 (4.24 ± 2.58)	36 (4.63 ± 2.11)	-0.769	0.444
*Days to Fever Occurrence*	*85 (2, 1 - 3)*	*49 (3, 2 - 4)*	*36 (1, 0 - 1)*	*1450*	*< 0.001*
*Fever Length*	*83 (4, 3.5 - 6)*	*48 (4, 3 - 5.25)*	*35 (5, 4 - 6)*	*566.5*	*0.01*
*Days to CRS Occurrence*	*84 (2, 1 - 3)*	*48 (3, 1.75 - 4)*	*36 (1, 0 - 1)*	*1414*	*< 0.001*
*CRS Length*	*84 (4, 3.75 - 6)*	*48 (4, 3 - 5.25)*	*36 (5.5, 4 - 6.25)*	*539*	*0.003*
Hospitalization Stay	95 (26, 20 - 38)	59 (25, 19 - 38.5)	36 (30.5, 22 - 36.5)	893	0.196
*Corticosteroids Medication, Yes or Not*					*<0.001*
*N*	64 (68.09)	53 (91.38)	11 (30.56)		
*Y*	30 (31.91)	5 (8.62)	25 (69.44)		
*Tocilizumab Medication, Yes or Not*					*<0.001*
*N*	22 (23.16)	21 (35.59)	1 (2.78)		
*Y*	73 (76.84)	38 (64.41)	35 (97.22)		
*Vasoactive Medication, Yes or Not*					*<0.001*
*N*	52 (54.74)	47 (79.66)	5 (13.89)		
*Y*	43 (45.26)	12 (20.34)	31 (86.11)		
*Ventilation, Yes or Not*					*<0.001*
*N*	81 (85.26)	58 (98.31)	23 (63.89)		
*Y*	14 (14.74)	1 (1.69)	13 (36.11)		
ICANS Category Dichotomized at Level 2 after CAR-T					0.151
*non-sICANS*	87 (91.58)	56 (94.92)	31 (86.11)		
*sICANS*	8 (8.42)	3 (5.08)	5 (13.89)		

^1^ Continuous variables in each group were displayed as “Count (Mean ± SD)” if corresponding normality test is passed, otherwise they were displayed as “Count (Median, Q25 ~ Q75)”. Whereas categorical variables were displayed as the number of participants with non-missing values and its proportion (%) with respect to all the participants with non-missing values in a given group.

^2^ Continuous variables were globally tested with ANOVA if the variables satisfy normal distribution in each subgroup, otherwise tested with Kruskal-Wallis test. The Shapiro normality test was implemented for testing the normality of these continuous variables. Categorical variables were tested with Fisher’s exact test.

### 3.2 Expression profiling of biomarkers following CAR-T therapy

**Figure 1B** illustrates the cytokine expression profiles observed in individual patients with or without sCRS. Among the cytokines examined, IL-6, IFN-γ, IL-10, and IL-2 showed a marked increase during the onset of sCRS. These trends are further demonstrated in **Figure 1C**, which presents a comparative analysis of cytokine expression patterns between sCRS and non-sCRS groups at three critical time points: before CAR-T cell infusion, fever onset, and peak CRS. The results show elevated IL-6, IFN-γ, IL-10, and IL-2 expression levels in sCRS patients spanning from ‘ before CAR-T ‘ to ‘ peak CRS ‘, which returned to normal levels after one week post-infusion. Notably, IL-2 reached its peak expression levels earlier than the other cytokines (3 days vs. 5-6 days). In contrast, no significant changes were observed in cytokine expression among non-sCRS or sCRS patients for TNF-α, IL-17A or IL-4 (**Supplementary Figure 1**). In addition, four routine markers (CRP, PCT, LDH and Ferritin) also showed elevated expression levels in sCRS patients following CAR-T infusion (**Figure 1D**). By comparison, the levels of hematological markers (WBC, Neutrophil, Platelet and Hemoglobin) were reduced in sCRS group compared to non-sCRS group except for lymphocyte counts which showed reduced expression level in sCRS group following CAR-T infusion before Day 5 while elevated expression level after Day 5 (**Figure 1E**). Finally, the statistical significance of each biomarker profile was analyzed specifically between the sCRS and non-sCRS groups, and the respective p-values have been incorporated into **Figures 1C–E**.

### 3.3 Comparation of biomarkers between non-sCRS group and sCRS group

**Figure 2:**
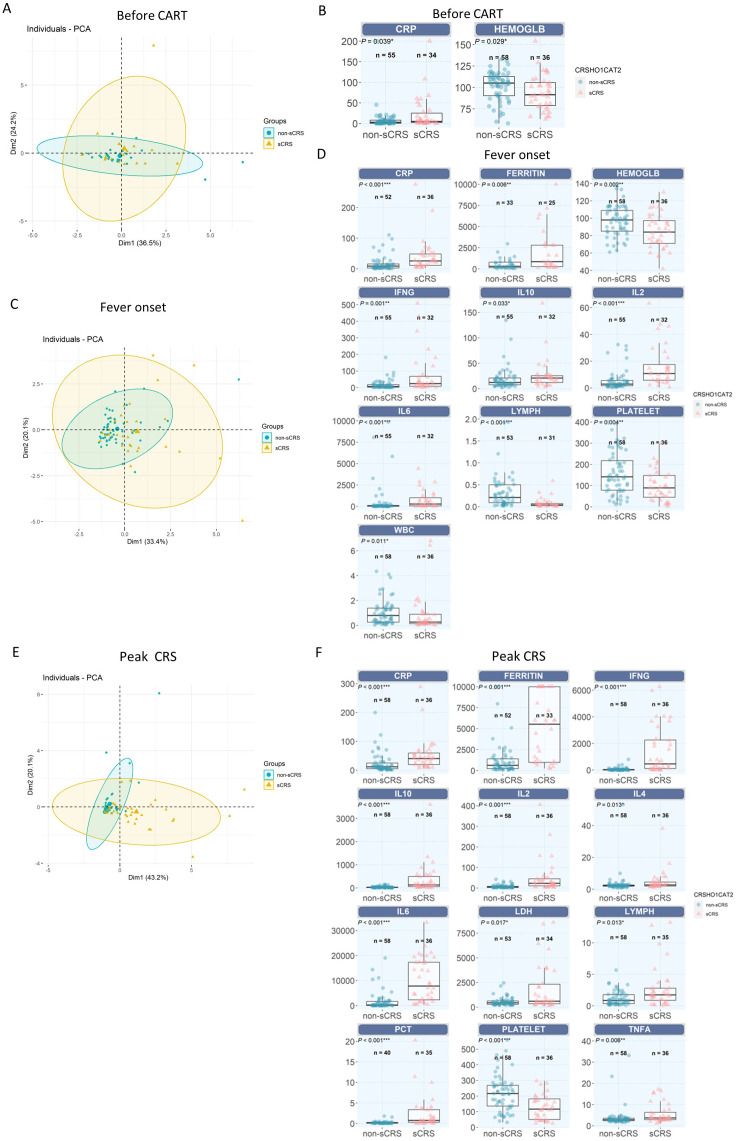
Comparation of different biomarkers between non-sCRS group and sCRS group. **(A, C)** and **(E)** Principal component analysis (PCA) model for the dimensionality reduction and visualization of the patients **(A)** before CAR-T infusion, **(C)** at fever onset and **(E)** at peak CRS. **(B, D, F)** Cytokine profiles were compared in patients who developed sCRS with patients who did not **(B)** before CAR-T infusion, **(D)** at fever onset and **(F)** at peak CRS. ***, p < 0.001, **, p < 0.01, *, p < 0.05.

To find the best biomarkers to distinguish the non-sCRS group and sCRS group, we applied the principal component analysis (PCA) model for the dimensionality reduction and visualization of the patients. As shown in **Figure 2A**, prior to CAR-T infusion, there is no significant separation between non-sCRS group and sCRS group. Then, we analyzed the differential expression of all the available biomarkers prior to CAR-T infusion and found there are only two biomarkers that have differential expression between these two groups, CRP (**Figure 2B**, p = 0.039) and hemoglobin (**Figure 2B**, P = 0.029). When comparing the non-sCRS and sCRS groups at fever onset, there was still no significant separation between non-sCRS group and sCRS group, but there was a trend of separation between these two groups (**Figure 2C**). And we found that four of the seven cytokines detected, IL-2 (P < 0.001), IL-6 (P < 0.001), IFN-γ (P = 0.001), and IL-10 (P =0.033) had increased expression in sCRS patients (**Figure 2D**). Additionally, two conventional biomarkers, CRP (P < 0.001) and Ferritin (P=0.006) were also increased in the sCRS group compared to the non-sCRS group (**Figure 2D**). Moreover, the sCRS group had lower levels of lymphocyte counts (P < 0.001), hemoglobin (P = 0.002), platelets (P = 0.004) and WBC counts (P = 0.011) compared to the non-sCRS group at fever onset (**Figure 2D**). Lastly, we compared the non-sCRS and sCRS groups at peak CRS and found that there was a significant separation between non-sCRS group and sCRS group (**Figure 2E**). And when comparing the differential expression of all the biomarkers at peak CRS, we found that, except for IL-17A, all cytokines exhibited increased expression in sCRS patients compared to non-sCRS patients (**Figure 2F**). Moreover, four conventional markers (CRP, PCT, Ferritin and LDH) were also increased in the sCRS group compared to the non-sCRS group (**Figure 2F**). In terms of hematological parameters, higher lymphocyte counts levels and lower platelet levels were observed in the sCRS group (**Figure 2F**).

### 3.4 Performance of biomarkers for sCRS prediction

**Figure 3:**
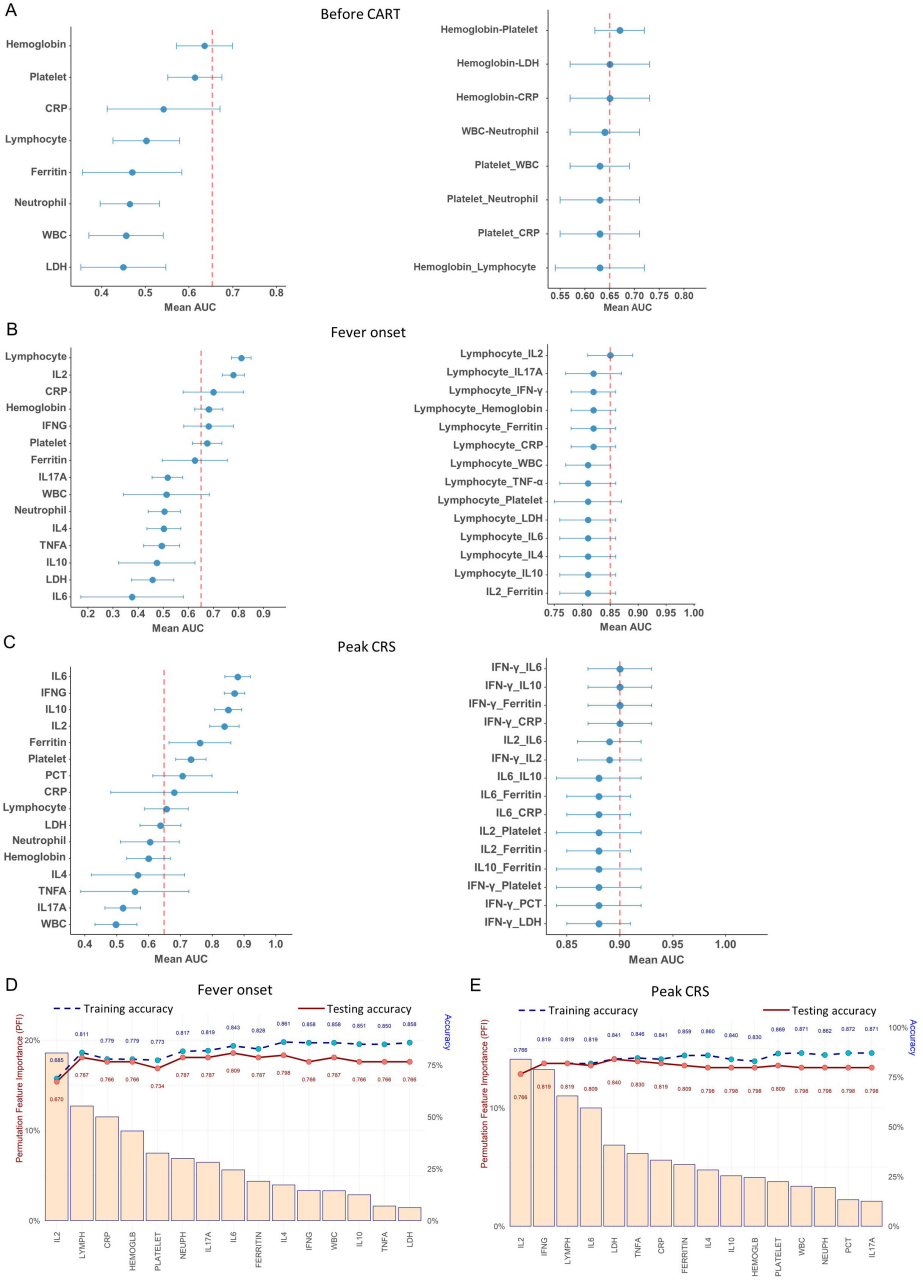
Performance of different biomarkers using the logistic regression. **(A–C)** Forest plots depicting AUC relative to each biomarker and two biomarkers combination for sCRS prediction **(A)**before CAR-T infusion, **(B)** at fever onset and **(C)** at peak CRS. **(D, E)** Feature importance for discrimination between non-sCRS group and sCRS group. Cytokines are listed in terms of decreasing feature importance (left axis), and the performance of the model is shown (training and testing accuracy) when predictors are added to the model one at a time (right axis) **(D)** at fever onset and **(E)** at peak CRS.

Next, we used cross-validated ROC to evaluate the performance of all the biomarkers at three important time windows. As shown in **Figure 3A**, prior to CAR-T infusion, the top three area under the curve (AUC) for predicting sCRS risk based on CRP, hemoglobin, and platelet levels was 0.54, 0.61, and 0.63, respectively and none of them with an AUC higher than 0.65. In addition, the various combinations of two different variables showed that only the combination of hemoglobin and platelet demonstrated the highest AUC (0.67), which suggests that the available laboratory tests showed limited information for prediction sCRS risk prior to CAR-T infusion (**Figure 3A**).

At fever onset, the lymphocyte counts (AUC = 0.81) and IL-2 (AUC = 0.78) were identified as the most effective biomarkers for predicting sCRS (**Figure 3B**). Furthermore, the combination of lymphocyte counts and IL-2 demonstrated the highest AUC (0.85) among the various combinations of two different variables (**Figure 3B**). However, incorporating other biomarkers with lymphocyte counts did not significantly enhance its performance compared to using lymphocyte counts (AUC = 0.81) alone (**Figure 3B**). To further assess the feature importance of the available biomarkers, we next utilized a machine-learning feature forward selection approach elastic net model with leave-one-out-cross validation (LOOCV) in our analysis. Our findings indicate that IL-2 is the primary contributor to the accuracy of early detection for sCRS (Permutation Feature Importance closed to 20%), followed by lymphocyte counts, and the addition of other variables could slightly enhance the accuracy (**Figure 3D**). All these results indicate that IL-2 is the most effective cytokine biomarker for detecting sCRS in its early stages among all the cytokines examined and the combination of IL-2 with hematological markers lymphocyte counts achieved the highest AUC for early detecting sCRS.

We then evaluated the performance of various biomarkers in predicting sCRS at peak CRS. As shown in **Figure 3C**, the AUC value was analyzed for each biomarker and it was found that IFN-γ, IL-6, IL-10, and IL-2 had the better AUC values (all above 0.8), with IL-6 having the highest value of 0.88. We then investigated different combinations of two variables and found that the combinations that included top-ranked proteins, especially IFN-γ, had strong AUC values. The combinations of IFN-γ plus IL-6/IL-10/Ferritin/CRP had the best performance, reaching an AUC of 0.90 (**Figure 3C**). When the analysis was performed using the elastic net model, it was determined that IL-2 was still the most important contributor to the accuracy of sCRS prediction, followed by IFN-γ (**Figure 3E**). When using IL-2 alone, we achieved an accuracy of 0.766 in predicting sCRS. Adding IFN-γ increased the accuracy to 0.819. When we further added other markers, the accuracy of the model increased further to about 0.87.

Overall, our findings suggest IL-2, IFN-γ, IL-6 and IL-10 emerge as the important cytokines for predicting the occurrence of sCRS. Levels of IL-2 rise at an earlier stage of sCRS and may potentially serve as the most effective biomarker for promptly detecting the onset of the condition. IFN-γ and IL-6 emerge as important cytokines at later stage. The elastic net model demonstrated that IL-2 is the top-ranking contributor to sCRS prediction model, either in fever onset or peak CRS scenarios. Moreover, a combination of these cytokine biomarkers with hematological or conventional markers may further increase their performance in predicting the occurrence of sCRS.

### 3.5 Predictive modeling for Early prediction of sCRS

**Figure 4:**
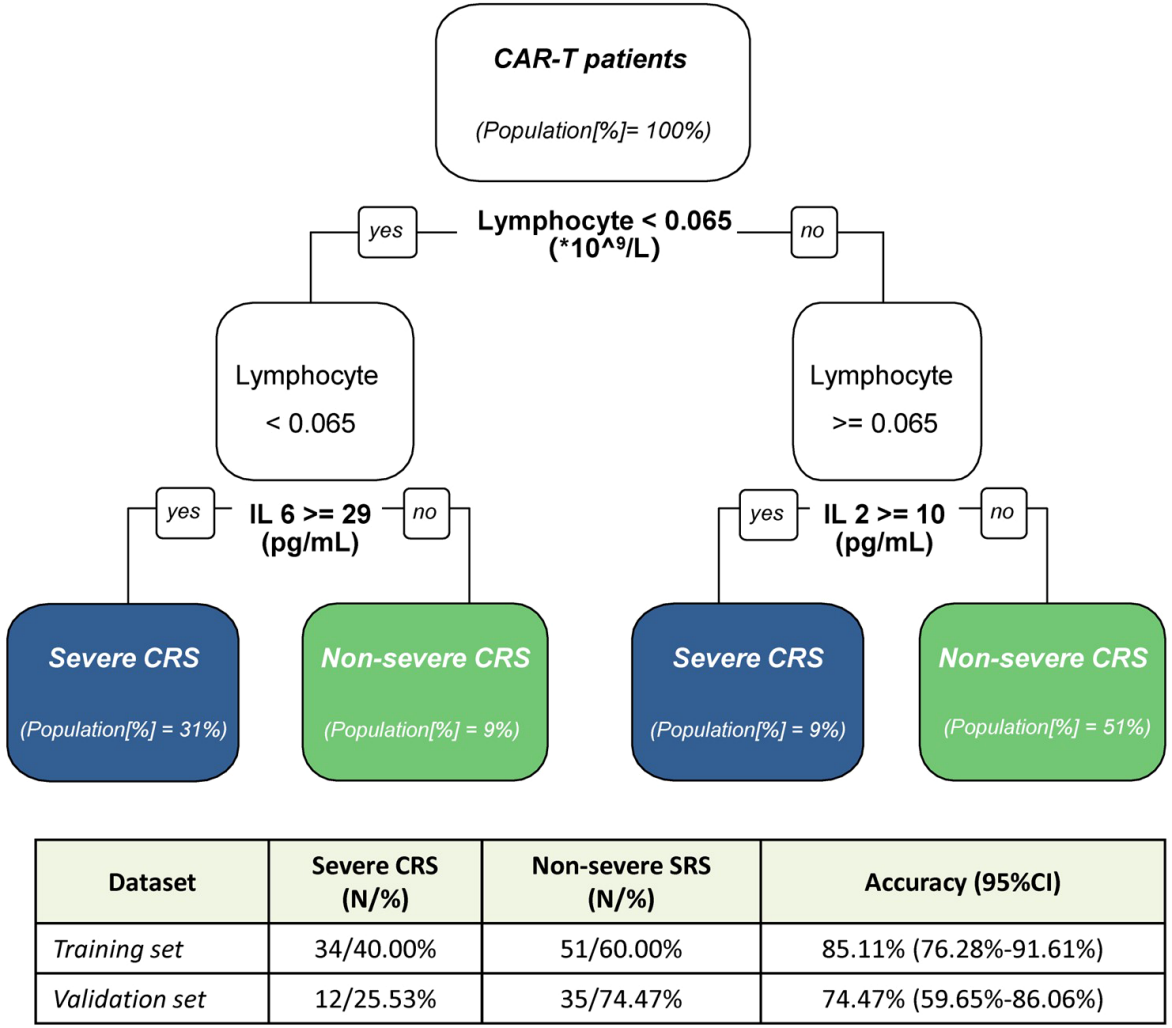
Decision tree modeling for early prediction of severe CRS. Decision tree model for discrimination between non-sCRS group and sCRS group. In the table, N represent the number of patients, and in the classification tree [%] indicate the proportion of patients from the training cohort. CI indicate the confidence interval.

After evaluating the performance of different biomarkers in predicting sCRS at three important time windows, we further investigated whether we could build a model to identify the patients who would subsequently develop life-threatening CRS early after CAR-T cell infusion so that intervention strategies that might prevent progression of CRS could be started. According to the results we have obtained about the performance of different biomarkers in predicting sCRS in the previous part, we performed the classification decision trees modeling and compared the accuracies of different biomarkers combinations models. A total of 85 patients were included in the training cohort, while 10 patients were excluded due to missing data for certain variables. In the end, lymphocyte counts, IL-2 and IL-6 were selected as the best classification model at fever onset. By using this model, patients with lymphocyte counts < 0.065 × 10^9^/L and IL-6 ≥ 29 pg/ml, or lymphocyte counts < 0.065 × 10^9^/L and IL-2 ≥ 10 pg/ml were classified as sCRS group while patients with lymphocyte counts ≥ 0.065×10^9^/L and IL-6 < 29 pg/ml, or lymphocyte counts ≥ 0.065 × 10^9^/L and IL-2 < 10 pg/ml were classified as non-sCRS group (**Figure 4**). This model achieved the cross-validated highest accuracy of 85.11% (95% CI = 0.763–0.916). Subsequently, we evaluated the predictive accuracy of this model in a validation cohort consisting of 47 pediatric patients with relapsed/refractory B-ALL who underwent CAR-T cell therapy. The characteristics of the patients in this cohort are summarized in **Supplementary Table 1**. Our findings indicated that the model exhibited satisfactory performance in the validation cohort, achieving an accuracy of 74.47% (95% CI = 0.597–0.861). Collectively, the presented data suggest that a synergistic combination of interleukin-2 (IL-2) and interleukin-6 (IL-6), coupled with lymphocyte counts, holds potential as early predictive biomarkers for sCRS.

## 4 Discussion

In this study, we investigated the clinical characteristics and laboratory test profiles of 95 pediatric patients with R/R B-ALL or B-NHL who received CD19- and CD22- targeted CAR-T cell therapy. We conducted a dynamic analysis of biomarker expression and identified early and peak biomarkers of severe CRS across various time windows. Notably, our findings highlighted an early surge in IL-2 levels following CAR-T cell infusion, suggesting that IL-2 may serve as an early predictor of severe CRS. At the onset of fever, we developed an early predictive model for severe CRS for the first time, incorporating readily accessible clinical parameters such as lymphocyte counts, IL-2, and IL-6 levels. Our data provide a novel perspective on the immunopathogenesis of CRS and may help physicians to manage CRS more effectively.

CRS is a direct consequence of overactivation of the immune system, causing significant increase in several serum cytokines (24). Previous studies summarized that commonly elevated biomarkers during CRS including IL-6, INF-γ, TNF-α, IL-10, CRP, ferritin, and IL-8, etc (12, 18, 25–30). IL-6 is believed to be the most important biomarkers for CRS, and cytokine blockade targeting the IL-6 pathway is the current standard of care for the treatment of CRS (31). In this study, we found that IL-6, IFN-γ and IL-10 show a consistently marked differential increase in the sCRS group compared to the non-sCRS group throughout the CRS process, which is consistent with previous reports (12, 18, 25–28, 32). Of note, in the early stage of CRS, we observed that IL-2 was the earliest cytokine to reach its peak level than others, indicating IL-2 maybe an optional early cytokine biomarker to predict sCRS. Indeed, in subsequent analyses, IL-2 showed good performance in different predictive models to predict the development of sCRS. Although several literatures have reported that IL-2 may be a biomarker for severe ICANS (14, 33, 34), few reports show its direct correlation with sCRS. In a study of 15 Chinese R/R ALL patients treated with CAR-T19, IL-6 is one of the most important biomarkers for CRS but paired peak serum levels of IL-2 levels were not associated with CRS (27). DT. Teachey et al. evaluated 43 cytokine profiles compared in patients who developed severe CRS and patients who did not and found peak values of IL-2 over the first month was not statistically different by CRS severity in adult patients (n=12) (13). We noticed that the sample size in those two studies were small and the time windows to analyze the cytokine levels were different from ours. In a recent study with 200 B-ALL patients received CD19-targeted CAR-T cells, IL-2 was observed to be one of the sCRS related factors (35). Consistent with our study, this study is focus on the early factors for sCRS prediction. As shown in our study, the cytokines with their peak values related to the severity of CRS are kind of different with the ones predict sCRS at early days of fever onset. It may be important to identify early biomarkers to forecast the development and severity of CRS during CAR-T cell therapy. Early prediction could provide physicians with an opportunity to risk-stratify patients for the development of severe CRS, allowing them to mitigate the development of severe CRS before the patients become critically ill (36).

Based on the data from the current study, a predictive tree model was developed to identify the occurrence of severe CRS during CAR-T cell therapy at fever onset. The model is based on various factors that are easily accessible in clinical settings and operationally feasible. The model includes three factors: lymphocyte counts, IL-6, and IL-2. Patients are classified as sCRS group when lymphocyte counts < 0.065 × 10^9^/L and IL-6 ≥ 29 pg/ml or IL-2 ≥ 10 pg/ml. Conversely, patients are classified as non-sCRS group when lymphocyte counts ≥ 0.065 × 10^9^/L and IL-6 < 29 pg/ml or IL-2 < 10 pg/ml. By classifying patients into high-risk (severe CRS group) and low-risk (non-severe CRS group) categories based on specific threshold values for these factors, physicians can better stratify patients and potentially intervene earlier to mitigate the development of severe CRS. The model achieved an accuracy of 85.11% (95% CI = 0.763–0.916) in our training cohort, and demonstrated equally impressive performance in the validation cohort, with an accuracy of 74.47% (95% CI = 0.597–0.861). The model’s high accuracy rates in both the training and validation cohorts, demonstrate its robustness and potential as a diagnostic tool. However, it is important to perform further validations across diverse CAR-T therapeutic approaches and in patients of varying ages to ensure the generalizability and applicability of these findings in a broader clinical context.

In conclusion, we have identified and characterized biomarkers that are associated with sCRS and can predict which patients are likely to develop sCRS before it happens. We present an easy-to-use, clinically significant predictive tree model to make an early prediction of sCRS after CAR-T cells infusion. As CAR-T therapies have become more common, these data may provide significant novel information to better manage potential associated toxicities.

## Data Availability

The original contributions presented in the study are included in the article/**Supplementary Material**. Further inquiries can be directed to the corresponding authors.
