# Supplementary material for: Identification of early predictive biomarkers for severe cytokine release syndrome in pediatric patients with chimeric antigen receptor T-cell therapy
Source: Front Immunol. 2024 Sep 12;15:1450173. doi: 10.3389/fimmu.2024.1450173 (PMC11424402; doi:10.3389/fimmu.2024.1450173)
Supplement: Supplementary file 1 [file DataSheet1.docx]

Supplementary Material

**Supplementary Appendix 1.**

**Inclusion criteria:**

Subjects with relapsed or refractory pediatric hematological malignancies, under the age of 18 at disease onset, who meet any of the following criteria:

a) Initial patients were identified as meeting the criteria for refractory disease following chemotherapy: Refractory B-ALL patients who did not achieve remission (>5% blasts in bone marrow) after two or more courses of remission induction treatment or those with refractory leukemia with minimal residual disease >1% at the end of remission induction and persistent >0.1% after consolidation treatment who are ineligible for allogeneic stem cell transplant (alloSCT); Refractory B-NHL patients who, after 4 courses of standard chemotherapy, show <50% tumor shrinkage or experience disease progression;

b) Disease progression on chemotherapy in patients with initial diagnosis and expected poor chemotherapy outcome;

c) Patients with one or more relapses with clear evidence of residual tumor;

d) Patients with disease recurrence after autologous or allogeneic hematopoietic stem cell transplantation;

e) Patients with hematological oncological diseases for which current surgical, radiotherapeutic and chemotherapeutic modalities offer no prospect of cure.

**Exclusion criteria:**

Subjects will not be included in the study if any of the following criteria applies:

a) Current autoimmune disease, or history of autoimmune disease with potential CNS involvement;

b) Active clinically significant CNS dysfunction (including but not limited to uncontrolled seizure disorders, cerebrovascular ischemia or hemorrhage, dementia, paralysis);

c) History of an additional malignancy other than non-melanoma skin cancer or carcinoma in situ unless disease free for >= 3 years;

d) Pulmonary function: Patients with pre-existing severe lung disease (FEV1 or FVC < 65%) or an oxygen requirement of >28% O2 supplementation or active pulmonary infiltrates on chest X-ray at the time scheduled for T cell infusion;

e) Cardiac function: Fractional shortening <28% or left ventricular ejection fraction < 50% by echocardiography;

f) Renal function: Creatinine clearance < 50 mL/min/1.73 m2;

g) Liver function: Patients with a serum bilirubin >3 times upper limit of normal or an AST or ALT > 5 times upper limit of normal, unless due to leukemic liver infiltration in the estimation of the investigator;

h) Rapidly progressive disease that in the estimation of the investigator would compromise ability to complete study therapy;

i) Active Hepatitis B (HBsAg positive) or Hepatitis C (PCR positive), or known infection with human immunodeficiency virus (HIV);

j) Committal to an institution on judicial or official order.


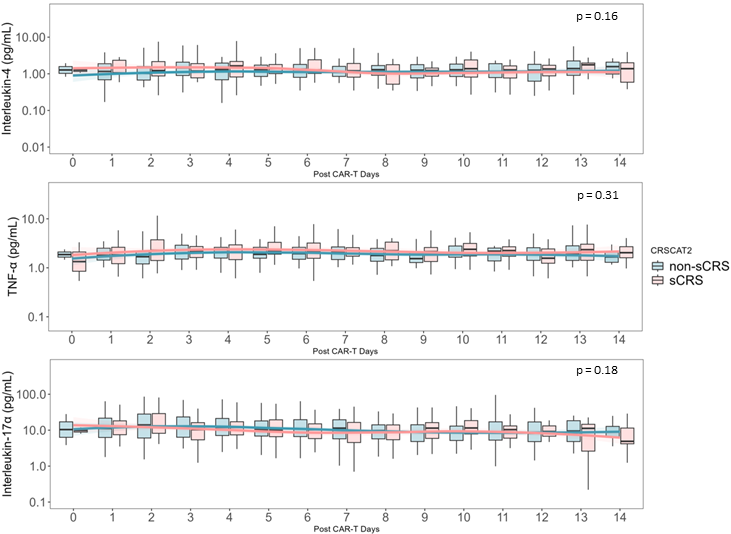


**Supplementary Figure 1. Cytokine expression patterns in non-sCRS group and sCRS group.** Concentrations of three released cytokines in patients at the indicated time points. Repeat measurement of mixed model were conducted to compare between group difference of the profiling of each biomarker during study period and p values between non-sCRS group and sCRS group were shown.

| **Supplementary Table 1. Baseline characteristics between different CRS groups in the Validation Cohort** | | | | | |
| --- | --- | --- | --- | --- | --- |
| **Variable** | **All Participants***^1^* | **CRS Grading***^1^* | | **Statistic** | ***P****^2^* |
|  |  | **Grade, non-sCRS (n = 35)** | **Grade, sCRS (n = 12)** |  |  |
| Age (Years) | 47 (8.4 ± 4.5) | 35 (8.35 ± 4.49) | 12 (8.56 ± 4.74) | -0.14 | 0.889 |
| Gender |  |  |  |  | 0.481 |
| *FEMALE* | 15 (31.91) | 10 (28.57) | 5 (41.67) |  |  |
| *MALE* | 32 (68.09) | 25 (71.43) | 7 (58.33) |  |  |
| Bone marrow blasts before CAR-T(%) | 47 (0.02, 0 - 0.07) | 35 (0.02, 0 - 0.05) | 12 (0.05, 0 - 0.16) | 180 | 0.472 |
| Days to CRS Occurrence | 40 (1, 1 - 3) | 28 (1, 1 - 3) | 12 (1, 1 - 2) | 188 | 0.527 |
| *CRS Length* | *41 (4.95 ± 2)* | *29 (4.55 ± 1.76)* | *12 (5.92 ± 2.27)* | *-2.069* | *0.045* |
| Corticosteroids Medication, Yes or Not |  |  |  |  | <0.001 |
| *N* | 33 (70.21) | 32 (91.43) | 1 (8.33) |  |  |
| *Y* | 14 (29.79) | 3 (8.57) | 11 (91.67) |  |  |
| *Tocilizumab Medication, Yes or Not* |  |  |  |  | *0.021* |
| *N* | 13 (27.66) | 13 (37.14) | 0 |  |  |
| *Y* | 34 (72.34) | 22 (62.86) | 12 (100) |  |  |
| ICANS Category Dichotomized at Level 2 after CAR-T |  |  |  |  | 0.156 |
| *non-sICANS* | 44 (93.62) | 34 (97.14) | 10 (83.33) |  |  |
| *sICANS* | 3 (6.38) | 1 (2.86) | 2 (16.67) |  |  |
| *^1^* Continuous variables in each group were displayed as "Count (Mean ± SD)" if corresponding normality test is passed, otherwise they were displayed as "Count (Median, Q25 ~ Q75)". Whereas categorical variables were displayed as the number of participants with non-missing values and its proportion (%) with respect to all the participants with non-missing values in a given group. | | | | | |
| *^2^* Continuous variables were globally tested with ANOVA if the variables satisfy normal distribution in each subgroup, otherwise tested with Kruskal-Wallis test. The Shapiro normality test was implemented for testing the normality of these continuous variables. Categorical variables were tested with Fisher's exact test. | | | | | |
